# Effect of the Red Bull Energy Drink on Perfusion-Related Variables in Women Undergoing Microsurgical Breast Reconstruction: Protocol and Analysis Plan for a Prospective, Multicenter Randomized Controlled Trial

**DOI:** 10.2196/38487

**Published:** 2023-05-09

**Authors:** Nicole Edith Speck, Michal Michalak, Kathrin Dreier, Doris Babst, Alessia Marisa Lardi, Jian Farhadi

**Affiliations:** 1 Plastic Surgery Group Zurich Switzerland; 2 Department of Plastic, Reconstructive, Aesthetic and Hand Surgery University Hospital Basel Basel Switzerland; 3 Department of Computer Science and Statistics Poznan University of Medical Sciences Poznan Poland; 4 Department of Anesthesiology Klinik Pyramide am See Zurich Switzerland; 5 Faculty of Medicine University of Basel Basel Switzerland

**Keywords:** analysis, blood pressure, breast, energy drinks, heart rate, mammaplasty, microsurgery, pain, patient, perfusion, postoperative, prospective studies, reconstruction, surgery, systolic, taurine

## Abstract

**Background:**

Maintaining a sufficiently high systolic blood pressure is essential for free flap perfusion after microsurgical breast reconstruction. Yet, many women undergoing these procedures have low postoperative systolic blood pressure. Intravenous volume administration or vasopressors may be needed to maintain systolic blood pressure above a predefined threshold. However, excessive volume administration may lead to volume overload and flap stasis, and the postoperative use of vasopressors may be limited depending on institutional standards. Additional nonpharmacological measures to raise blood pressure might be beneficial. Evidence suggests that the Red Bull energy drink could raise blood pressure. It has been shown to increase systolic and diastolic blood pressure in healthy volunteers and athletes.

**Objective:**

The primary objective of this study is to determine the difference in systolic blood pressure between an intervention group receiving Red Bull and a control group receiving still water after microsurgical breast reconstruction. Secondary objectives include postoperative heart rate, 24-hour fluid balance, pain level, or necessity for revision surgery due to flap complications.

**Methods:**

The Red Bull study is a prospective, multicenter randomized controlled trial comparing the effect of postoperative ingestion of Red Bull energy drink against still water in female patients undergoing unilateral microsurgical breast reconstruction. A total of 250 mL of Red Bull (intervention group) or 250 mL of still water (control group) will be administered to the study participants 2 hours postoperatively as well as for breakfast and lunch on postoperative day 1, amounting to a total volume of 750 mL per 24 hours. Female patients between 18 and 70 years of age undergoing unilateral microsurgical breast reconstruction will be included. Exclusion criteria are a history of arterial hypertension, cardiac rhythm disorder, diabetes mellitus, gastric or duodenal ulcer, thyroid disease, and current use of antihypertensive or antiarrhythmic drugs or thyroid hormones, as well as intolerance to Red Bull.

**Results:**

Recruitment for the study started in June 2020 and was completed in December 2022. There is evidence that the Red Bull energy drink increases blood pressure in healthy volunteers and athletes. We hypothesize that postoperative ingestion of Red Bull will increase systolic blood pressure in women after microsurgical breast reconstruction. Red Bull could hence be used as a nonpharmacological adjunct to vasopressors or volume administration in women with hypotensive blood pressure after microsurgical breast reconstruction.

**Conclusions:**

This paper describes the Red Bull study trial protocol and analysis plan. The information will increase the transparency of the data analysis for the Red Bull study.

**Trial Registration:**

ClinicalTrials.gov NCT04397419; https://clinicaltrials.gov/ct2/show/NCT04397419

**International Registered Report Identifier (IRRID):**

DERR1-10.2196/38487

## Introduction

### Overview

Maintaining a sufficiently high systolic blood pressure is vital for free flap perfusion after microsurgical breast reconstruction. However, many women undergoing microsurgical breast reconstruction have low systolic blood pressure postoperatively. Often, intravenous volume administration or vasopressors are needed to maintain systolic blood pressure above 100 mm Hg. Yet, intravenous volume administration may lead to volume overload and flap stasis, and the use of vasopressors in the postoperative setting may be limited depending on institutional policies. Additional nonpharmacological measures to guarantee adequate postoperative perfusion pressure would be beneficial.

### Clinical Evidence for Red Bull Energy Drink

Red Bull energy drink has shown varying effects on the hemodynamic profile and pain perception in clinical studies performed on healthy participants and athletes [1-3]. A randomized crossover study showed a negative hemodynamic profile in response to Red Bull [2]. Participants who ingested 355 mL of Red Bull showed increased systolic and diastolic blood pressure, increased heart rate, and cardiac output without significantly reduced endothelial function compared with a control group who received 355 mL of tap water. This was reproduced in another randomized crossover study that further explored the combined effect of Red Bull and mental stress on cardiovascular parameters [1]. The combination of Red Bull and mental stress leads to an additional increase in systolic and diastolic blood pressure and heart rate, suggesting a cumulative effect.

Energy drinks have shown varying effects on endothelial function [4,5]. In a case report, a reduced reactive hyperemia index was observed after ingestion of Monster energy drink, suggesting worsened endothelial function [4]. In contrast, Red Bull improved endothelial function in a prospective study involving 6 volunteers [5]. Ingestion of 250 mL of Red Bull significantly increased endothelial function as measured by the reactive hyperemia index. In the same study, coffee containing comparable amounts of caffeine did not affect endothelial function. These results suggest that ingredients other than caffeine account for the vasodilatory effect.

Considering the mild diuretic effect of caffeine, Red Bull energy drink may increase urine formation rate and decrease urine specific gravity. Although urinary output increased with caffeine and Red Bull energy drink in 1 study [6], this trend could not be observed elsewhere [7].

Red Bull energy drink may furthermore increase pain tolerance [7]. Levels of pain after a cold pressor test were measured in participants before and after consumption of 250 mL of Red Bull. All participants reported a decreased perception of pain directly following Red Bull consumption.

### Interventional Product

Red Bull energy drink was first introduced in Austria in 1987 and launched in Switzerland in 1994. It is among the most popular energy drinks with over 6 billion cans being sold annually worldwide [8]. A can of 250 mL Red Bull energy drink contains 80 mg caffeine, 1 g taurine, 240 mg glucuronolactone, 27 g carbohydrates as well as water, sodium citrate, vitamins (B3, B5, B6, and B12), flavoring, and coloring agents [9]. This corresponds to total calorie content of 194 kJ (46 kcal) per 100 mL [8].

Despite anecdotal reports that linked Red Bull consumption with ventricular tachycardia and symptoms of cardiovascular disease, limited daily consumption of 500 mL of Red Bull appears safe [7,9]. When investigated in clinical studies no deleterious effects of Red Bull energy drink on cardiovascular function have been found [10]. Any adverse effects after excessive consumption are most likely due to the accumulation of caffeine, which is known to cause nervousness, anxiety, tremor, insomnia, tachycardia, arrhythmia, and nausea with ingestion exceeding 200 mg/day [11,12]. The amounts of taurine contained in Red Bull are far below the threshold to cause adverse effects [11].

Caffeine is an adenosine receptor antagonist with stimulating properties on the central nervous system It is known to increase blood pressure and peripheral vascular resistance through sympathetic stimulation [13]. Taurine is a sulfur-containing amino acid that constitutes the most abundant intracellular amino acid in humans [14]. Taurine seems to modulate skeletal muscle contractile function and possesses antiarrhythmic, inotropic, and chronotropic properties [15]. Glucuronolactone occurs naturally in small amounts within the body. Little data exists on its function in humans [9]. The amount of sugar provided in a can of 250 mL Red Bull energy drink is 27 g, which corresponds to 6.5 teaspoons of sugar [9]. Consuming moderately concentrated carbohydrate solutions (5%-8%) has been shown to promote prolonged exercise performance without causing adverse effects [15].

### Dose Rationale

The authors selected a daily administration of 250-500 mL of Red Bull in the study. Most investigators studied the effect of Red Bull after ingestion of 250, 355, or 500 mL. When ingested in standard amounts, Red Bull energy drink is a safe beverage without notable side effects [7,9].

### Risks

Daily oral administration of 500 mL of Red Bull has proven safe, and any side effects after consumption exceeding 500 mL daily have been linked to caffeine [9]. These include nervousness, anxiety, tremor, insomnia, tachycardia, arrhythmia, and nausea. To minimize adverse events, participants with allergies or intolerance to any component of Red Bull will be excluded from the study.

### Study Aim

The authors prospectively study the effect of postoperative ingestion of Red Bull energy drink on perfusion-related variables and patient recovery after microsurgical breast reconstruction. To our knowledge, there are no competing trials.

### Objectives

This study aims to assess perfusion-related variables, postoperative levels of pain, type of postoperative complications, operative reexploration rate due to complications, and total duration of hospital stay in patients undergoing autologous microsurgical breast reconstruction with postoperative ingestion of Red Bull Energy drink.

#### Primary Objective

The primary objective is to assess the difference in systolic blood pressure (mm Hg) between the intervention group and the control group at 15, 30, 45, 60, 75, 90, 105, and 120 minutes after each of 3 Red Bull (intervention group) or water (control group) applications.

#### Secondary Objective

The secondary objective is to document and assess the following variables in the intervention group and the control group:

1. Diastolic and mean arterial blood pressure (mm Hg) at 15, 30, 45, 60, 75, 90, 105, and 120 minutes after each of 3 Red Bull (intervention group) or water (control group) applications.
2. Postoperative heart rate (1/min) at 15, 30, 45, 60, 75, 90, 105, and 120 minutes after each of 3 Red Bull (intervention group) or water (control group) applications.
3. Postoperative urinary output (mL) during the first postoperative 24 hours.
4. Intraoperative and postoperative fluid administration (mL) until 24 hours after surgery.
5. Intraoperative and postoperative administration of vasoactive drugs (mg) until 24 hours after surgery.
6. Postoperative pain (numeric rating scale [NRS]) during the first postoperative 24 hours.
7. Postoperative administration of opioid analgesics (analgesic in mg).
8. Postoperative administration of nonopioid analgesics (analgesic in mg).
9. Postoperative administration of benzodiazepines (mg).
10. Rate of operative reexplorations during the first hospitalization (%).
11. Flap loss (%).
12. Duration of hospital stay (number of nights spent at hospital).
13. Wound healing 4 weeks after surgery (photo documentation).

#### Safety Objective

Notable adverse events of Red Bull energy drink in this population are as follows: nervousness, nausea, anxiety, tremor, insomnia, arrhythmia, need for revision surgery, and flap loss.

## Methods

### Overview

This is an open-label, multicenter, prospective, and randomized controlled trial comprising a cohort of 100 patients. Patient selection and inclusion as well as the anesthetic visit will occur preoperatively in an outpatient clinic. During hospitalization, microsurgical breast reconstruction will be performed under general anesthesia by JF, AML, or DB. Additionally, oral ingestion of Red Bull energy drink (intervention group) or still water (control group) will occur 2 hours after surgery and on a postoperative day 1 for breakfast and for lunch. Finally, wound healing and scarring will be assessed at the 4-week follow-up in our clinic using photo documentation.

Participants are expected to participate from the signing of the informed consent form until the 4-week follow-up visit. All patients enrolled prospectively in the trial will receive a Red Bull energy drink or still water. Blinding or masking is not feasible.

### Study Population

The study population will be women between 18 and 70 years of age undergoing unilateral microsurgical breast reconstruction by JF, AML, or DB at 2 institutions.

### Inclusion and Exclusion Criteria

Textbox 1 shows the inclusion and exclusion criteria.

As a standard of care, all patients are asked to perform a check-up by their general practitioner (GP) within 2-4 weeks before the microsurgical procedure. Their GP fills in a standardized form provided by the Institute of Anesthesiology, from which all inclusion and exclusion criteria can be drawn. Additionally, the GP performs an electrocardiogram and a blood test (complete blood count, electrolytes, creatinine, and international normalized ratio or Quick) for the patient and sends the result to the institute of anesthesiology at the respective clinic.

**Inclusion and exclusion criteria.**

**Inclusion criteria**

- Female patients between 18 and 70 years of age.
- Ability to give written informed consent before participation in the study.
- Ability to comply with the requirements and restrictions listed in the consent form.

**Exclusion criteria**

- History of arterial hypertension.
- History of cardiac rhythm disorder.
- History of diabetes mellitus.
- History of gastric or duodenal ulcer.
- History of hyperthyroidism or hypothyroidism.
- Current use of antihypertensive drugs, antiarrhythmic drugs, and thyroid hormones.
- Allergy or intolerance to the component of Red Bull energy drink.
- Not fulfilling inclusion criteria.

### Intervention

All consecutive female patients receiving unilateral autologous microsurgical breast reconstruction will be administered a total of either 750 mL Red Bull energy drink (intervention group) or 750 mL of still water (control group) at predefined intervals. The first dose of 250 mL of Red Bull energy drink or still water will be given 2 hours after surgery in the postanesthesia care unit. The following doses will be given on postoperative day 1 at 8 AM for breakfast (250 mL) and at 12 PM for lunch (250 mL). Full consumption of the study drink will be verified by a nurse. The study will be carried out at the 2 centers where JF, AML, and DB have been accredited: Klinik Pyramide am See, Zurich, Switzerland and Klinik Hirslanden, Zurich, Switzerland. The perioperative protocols have been standardized at both institutions and standard operating procedures have been implemented.

The systolic and diastolic blood pressure as well as pulse will be measured noninvasively using a 24-hour ambulatory blood pressure monitor (Tonoport VI, Anandic Medical Systems AG) during the first postoperative 24 hours. The device will be applied to the participant’s arm upon their arrival in the postanesthesia care unit and remain in place for 24 hours. Upon completion of these 24 hours, the readings will be downloaded on the study computer by the study personnel, using the software provided by Anandic Medical Systems AG.

Total fluid administered intraoperatively and postoperatively as well as intraoperative and postoperative administration of vasoactive drugs will be recorded. The daily urinary output will be measured 24 hours after surgery. Pain levels will be measured on NRS with values ranging from 0 to 10. The rate of operative reexplorations, flap loss, and total length of primary hospital stay will be recorded. Wound healing and scarring will be assessed 1 month after surgery in our clinic and documented photographically.

The variables obtained from the intervention group receiving Red Bull energy drink postoperatively will be compared with the corresponding variables of patients in the control group receiving the same amount of still water.

### Randomization and Blinding

Randomization is undertaken with equal allocation to each study group (Red Bull:still water=1:1) using a computed code. Blinding or masking is not feasible.

### Other Measures of Minimizing Bias

We will measure the systolic and diastolic blood pressure using ambulatory 24-hour blood pressure monitors (Tonoport VI, Anandic Medical Systems AG) to ensure comparable measurements (same side of arm and same cuff) and reduce intermeasurer variability. All patients will remain in bed during the first 12 hours postoperatively and will have a urinary catheter during this period. This further reduces changes in blood pressure not related to the study intervention. Additionally, all patients entering hospital preoperatively will have fasted for 6 hours (solids) and 2 hours (liquids). Also, oral fluid and food intake during the first 24 hours after a microsurgical procedure is typically low among all patients to ensure that patients could be taken back to theater in a timely manner should flap complications occur. The preoperative regular consumption of caffeinated products and the consumption of additional caffeinated products during the hospital stay will be recorded and incorporated in the final analysis. If a patient requires additional vasopressors postoperatively, this will be documented and incorporated in the final analysis.

### Outcomes

#### Primary Outcome

Difference in systolic blood pressure between the intervention group and control group at 15, 30, 45, 60, 75, 90, 105, and 120 minutes after each of 3 Red Bull (intervention group) or water (control group) ingestions as measured by the ambulatory blood pressure monitoring device.

#### Secondary Outcomes

Difference in the following postoperative variables between participants with or without postoperative intake of Red Bull energy drink:

- Diastolic and mean arterial blood pressure at 15, 30, 45, 60, 75, 90, 105, and 120 minutes after each of 3 Red Bull (intervention group) or water (control group) ingestions as measured by the ambulatory blood pressure monitoring device.
- Postoperative heart rate (1/min) at 15, 30, 45, 60, 75, 90, 105, and 120 minutes after each of 3 Red Bull (intervention group) or water (control group) applications.
- Postoperative urinary output (mL) during the first postoperative 24 hours.
- Intraoperative and postoperative fluid administration (mL) until 24 hours after surgery.
- Intraoperative and postoperative administration of vasoactive drugs (mL) until 24 hours after surgery.
- Postoperative pain (NRS) during the first postoperative 24 hours.
- Rate of operative reexplorations during the first hospitalization (%): flap complications (%) and flap loss (%); total duration of hospital stay (number of nights spent at hospital); wound healing 4 weeks after surgery (photo documentation).

#### Other Outcomes of Interest

Patient- and tumor-related data, such as age, BMI (size and weight), tobacco use; diabetes mellitus, indication for surgery (oncologic or prophylactic), neoadjuvant chemotherapy, previous irradiation, side and type of flap, flap weight, and ischemia time will be assessed perioperatively or collected from the medical history.

#### Harm Outcomes

Differences in the incidence of notable adverse events between both groups. Adverse events include nervousness, nausea, anxiety, tremor, insomnia, arrhythmia, need for revision surgery, and flap loss.

### Frequency and Duration of Follow-up

The schedule of trial enrolment, interventions, and assessments are presented in Table 1. Following surgery, the study intervention will take place in the recovery room and on the ward of the 2 study sites. The follow-up ends 4 weeks post operation in clinic.

**Table 1:** Schedule of enrolment, interventions, and assessments for the Red Bull study.

	First clinic appointment with the reconstructive team (preoperative)	Preoperative appointment with an anesthesiologist (up to 4 weeks before surgery)	Perioperative (treatment) period				Follow-up (4 weeks post surgery)
			Day of surgery	POD1^a^	Discharge from hospital		
Visit	1 (start)	2	3	3	3		4 (end)
Patient information and signed informed consent^b^	✓						
Demographic data	✓						
Medical history and letter from GP^c^	✓	✓					
Inclusion and exclusion criteria	✓						
Physical examination	✓						
Vital signs		✓ (provided by GP)	✓	✓			
Laboratory tests		✓ (provided by GP)					
Surgery (unilateral microsurgical breast reconstruction)			✓				
Administer Red Bull (intervention) or still water (control)			✓	✓			
Record blood pressure and heart rate			✓	✓			
Record urinary output			✓	✓			
Record volume of intravenous fluid administered			✓	✓			
Record volume of vasoactive drugs administered			✓	✓			
Record volume of oral fluid administered			✓	✓			
Record flap complications			✓	✓	✓		
Record operative reexploration			✓	✓	✓		
Record pain levels (NRS^d^ 0-10)			✓	✓	✓		
Record duration of hospital stay					✓		
Record adverse events of Red Bull ingestion			✓	✓	✓		
Assess wound healing and document photographically						✓	

^a^POD1: postoperative day.

^b^Informed consent form to be signed within 30 days of planned surgical procedure.

^c^GP: general practitioner.

^d^NRS: numeric rating scale.

### Recruitment

Participants are recruited for the study in outpatient clinic. All patients undergoing elective microsurgical breast reconstruction will be screened. Patients who consent to participate in the study are randomized at the moment of surgery planning. No compensation or payment is given to the study participants.

### Data Collection

Data are collected from clinical proformas, clinical notes, and study-specific forms and then entered onto a Excel (Microsoft Corp) file. The data collection form is provided in the Multimedia Appendix 1. The database has been programed by NES and MM. No identifiable data will be entered into the database. Participants will be identified using a unique code and initials. To avoid unauthorized people accidentally or deliberately perform changes, copies, or deletions of the analyzed data in Microsoft Excel, the following precautions are planned: after each relevant processing step, the new data version is saved and is not allowed to be changed. Such a new data version will be converted into a secured document (PDF file).

### Sample Size

The following assumptions estimated from literature and clinical experience:

- Mean systolic blood pressure (mm Hg): We assume the same SDs (ie, 15) for both groups and the correlation coefficient among repeated measures to be 0.7. If we assume no differences in time in the control group (mean 115 mm Hg) and assume the difference between the intervention group and the control group to be about 10 mm Hg, the required sample size would be 27 per group.
- Heart rate (1/min): We assume the same SDs (10) for both groups and the correlation among repeated measures to be 0.7. If we assume no differences in time in the control group (mean 80/min) and assume the difference between the intervention group and the control group to be 5/min, the required sample size would be 46 per group.
- Vasoactive drug Ephedrine (mg): We assume the mean drug dose in the control group to be 63 and the mean drug dose in the intervention group to be 48. Assuming equal SDs (ie, 25) we get 45 per group.

The predefined parameters include a significance level (α level) of .05 and power (1-β) of 0.8.

We calculated the patients needed to be included using the application STATA (version 15.1; StataCorp, 2017; Stata Statistical Software: Release 15) and G*Power (version 3.1.9.4—a program written by Franz Faul, Universität Kiel). Considering the different calculations and accounting for potential missing data or dropouts, 50 patients per group will be included, leading to a total of 100 patients.

### Statistical Methods

We are planning to measure mean systolic blood pressure at 8 time points. For this, the right statistical model is a mixed ANOVA where the group is an independent (unpaired) factor, and the time is a dependent (paired) factor. The comparison of drug intake between the Red Bull group and the control group will be performed by student *t* test for independent data (assuming data will follow a normal distribution). In case data will not follow a normal distribution, an alternative nonparametric test will be used (Mann-Whitney *U* test).

### Analysis Plan

As the null hypothesis (H0), we assume that mean systolic blood pressure from baseline to 120 minutes post drinking does not differ between the 2 groups. The analysis will be conducted by a senior statistician (MM) in accordance with the above mentioned statistical methods.

### Analysis of Harm Outcomes

The frequency of adverse events and the number of patients experiencing events will be tabulated by relation to the study intervention (ie, as adverse reaction, serious adverse event, or serious adverse reaction) and by study group. Nonserious adverse events unrelated to the intervention are recorded in the medical notes only.

### Handling of Missing Data and Dropouts

If vital data relevant for the calculation of the primary and secondary end point are missing (ie, blood pressure recordings) patients will be excluded from the participant list. Dropouts will be replaced by consecutive patients.

### Ethics Approval

Ethics approval was granted from the Cantonal Research Committee, Switzerland (KEK-ZH 2020-00493). Patients receive an information sheet and provide informed consent before screening to participate in study-specific procedures. The study will be carried out in accordance with the protocol and with principles enunciated in the current version of the Declaration of Helsinki, the guidelines of Good Clinical Practice issued by ICH, the Swiss Law and Swiss regulatory authority’s requirements.

### Compensation

Participants will not be compensated for their participation in this scientific research project.

### Publication and Dissemination Policy

After the statistical analysis of this trial, the principal investigator will make every endeavor to publish the data in a medical journal. Participants will be informed about the outcome of the study once the trial results have been published.

## Results

Recruitment of the study participants started in June 2020 and was completed in December 2022. As described previously, there is evidence that Red Bull energy drink increases blood pressure in healthy volunteers and athletes [1,2]. We hypothesize that systolic blood pressure will be higher after each drink ingestion in the intervention group receiving Red Bull compared with the control group receiving still water. Red Bull could hence be used as a nonpharmacological adjunct to vasopressors or volume administration after microsurgical breast reconstruction. It could be particularly useful in settings where the postoperative use of vasopressors is limited.

## Discussion

The Red Bull study is a multicenter randomized controlled trial that will explore the effect of Red Bull energy drink intake on perfusion-related variables and flap outcome after microsurgical breast reconstruction. This paper describes the study protocol and statistical analysis plan. The trial sponsor is Plastic Surgery Group. Recruitment started on June 1, 2020, and completed in December 2022. Database lock and data extraction for analysis were carried out in February 2023 when all randomized patients completed the surgical procedure and follow-up period.

There is evidence that the Red Bull energy drink increases blood pressure in healthy volunteers and athletes [1,2]. We hypothesize that Red Bull will increase systolic blood pressure in women after microsurgical breast reconstruction. Red Bull could hence be used as a nonpharmacological adjunct to vasopressors or volume administration in women with hypotension after microsurgical breast reconstruction.

### Strengths and Limitations

This trial has some strengths. This is the first trial assessing the influence of Red Bull on perfusion-related variables after surgery. To minimize adverse events and limit premature dropout from the study, participants with allergies or intolerance to any component of Red Bull will be excluded from the study. Also, diabetes mellitus is considered an exclusion criterion. Red Bull contains 27.5 g of sugar per 250 mL. Patients with diabetes mellitus might require the intake of sugar-free Red Bull, which could, however, be associated with a different hemodynamic pathway [16]. Furthermore, patients with thyroid disease or patients requiring the intake of thyroid hormones will be excluded from this study as the intake of Red Bull was associated with an increased heart rate in a previous study [2]. This trial also has some limitations. Blood pressure is measured noninvasively, which might not be as precise as arterial blood pressure monitoring.

After the statistical analysis of this trial, the principal investigator will make every endeavor to publish the data in a medical journal. Participants will be informed about the outcome of the study once the trial results have been published. The reporting of results from the outlined statistical analysis will comply with the consolidated standards of reporting trials protocol.

## Appendix

Multimedia Appendix 1 Data Collection Form.

## Abbreviations

GP: general practitioner
NRS: numeric rating scale

## Data Availability

The protocol and statistical analysis plan can be obtained by contacting the corresponding author. The data will be the property of the principal investigator. The study team will retain the exclusive use of the trial data until publication of major outputs has been completed.
