# Supplementary material for: Effect of the Red Bull Energy Drink on Perfusion-Related Variables in Women Undergoing Microsurgical Breast Reconstruction: Protocol and Analysis Plan for a Prospective, Multicenter Randomized Controlled Trial
Source: JMIR Res Protoc. 2023 May 9;12:e38487. doi: 10.2196/38487 (PMC10206609; doi:10.2196/38487)
Supplement: Multimedia Appendix 1 [file resprot_v12i1e38487_app1.docx]

**Case Report Form (CRF)**

| Studienort:  ☐ Klinik Pyramide am See  ☐ Klinik Hirslanden Zürich  Group: ☐ Red Bull® ☐ Water | Date of Surgery  ☐☐☐☐-☐☐-☐☐  (YYYY-MM-DD) |
| --- | --- |
|  | Date of Discharge  ☐☐☐☐-☐☐-☐☐  (YYYY-MM-DD)  Duration of Stay  ☐ nights |

| Year of birth ☐☐☐☐ | Age at surgery ☐☐ years |
| --- | --- |

| **Demography** | | |
| --- | --- | --- |
| Height | ☐.☐☐ [m] | |
| Weight | ☐☐☐ [kg] | |
| BMI | ☐☐.☐ [kg/m^2^] | |
| Smoker | yes ☐ no ☐ | PY: ☐☐ |
| Diabetes mellitus (Type 1 or Type 2) | yes ☐ no ☐ | |

| **Surgery** | |
| --- | --- |
| Breast cancer | yes ☐ no ☐ |
| Side of breast cancer | left ☐ right ☐ |
| Side of breast surgery | left ☐ right ☐ |
| Side of flap | left ☐ right ☐ |
| Flap type | DIEP ☐ SIEA ☐SGAP ☐  TMG ☐ TRAM ☐  Latissimus dorsi ☐  Other (specify) ☐ ____________________ |

| **Outcomes 1: hemodynamics** | |
| --- | --- |
| Baseline pre-operative blood pressure | ☐☐☐/☐☐☐ [mmHg] |
| Side measured (the same for all measurements) | right arm ☐ left arm ☐ |
| **After 1^st^ ingestion: time** |  |
| Systolic and diastolic blood pressure 15min after drink ingestion | ☐☐☐/☐☐☐ [mmHg] |
| Systolic and diastolic blood pressure 30min after drink ingestion | ☐☐☐/☐☐☐ [mmHg] |
| Systolic and diastolic blood pressure 45min after drink ingestion | ☐☐☐/☐☐☐ [mmHg] |
| Systolic and diastolic blood pressure 60 after drink ingestion | ☐☐☐/☐☐☐ [mmHg] |
| Systolic and diastolic blood pressure 75min after drink ingestion | ☐☐☐/☐☐☐ [mmHg] |
| Systolic and diastolic blood pressure 90min after drink ingestion | ☐☐☐/☐☐☐ [mmHg] |
| Systolic and diastolic blood pressure 105min after drink ingestion | ☐☐☐/☐☐☐ [mmHg] |
| Systolic and diastolic blood pressure 120min after drink ingestion | ☐☐☐/☐☐☐ [mmHg] |
| **After 2^nd^ ingestion: time** |  |
| Systolic and diastolic blood pressure 15min after drink ingestion | ☐☐☐/☐☐☐ [mmHg] |
| Systolic and diastolic blood pressure 30min after drink ingestion | ☐☐☐/☐☐☐ [mmHg] |
| Systolic and diastolic blood pressure 45min after drink ingestion | ☐☐☐/☐☐☐ [mmHg] |
| Systolic and diastolic blood pressure 60 after drink ingestion | ☐☐☐/☐☐☐ [mmHg] |
| Systolic and diastolic blood pressure 75min after drink ingestion | ☐☐☐/☐☐☐ [mmHg] |
| Systolic and diastolic blood pressure 90min after drink ingestion | ☐☐☐/☐☐☐ [mmHg] |
| Systolic and diastolic blood pressure 105min after drink ingestion | ☐☐☐/☐☐☐ [mmHg] |
| Systolic and diastolic blood pressure 120min after drink ingestion | ☐☐☐/☐☐☐ [mmHg] |
| **After 3^rd^ ingestion: time** |  |
| Systolic and diastolic blood pressure 15min after drink ingestion | ☐☐☐/☐☐☐ [mmHg] |
| Systolic and diastolic blood pressure 30min after drink ingestion | ☐☐☐/☐☐☐ [mmHg] |
| Systolic and diastolic blood pressure 45min after drink ingestion | ☐☐☐/☐☐☐ [mmHg] |
| Systolic and diastolic blood pressure 60 after drink ingestion | ☐☐☐/☐☐☐ [mmHg] |
| Systolic and diastolic blood pressure 75min after drink ingestion | ☐☐☐/☐☐☐ [mmHg] |
| Systolic and diastolic blood pressure 90min after drink ingestion | ☐☐☐/☐☐☐ [mmHg] |
| Systolic and diastolic blood pressure 105min after drink ingestion | ☐☐☐/☐☐☐ [mmHg] |
| Systolic and diastolic blood pressure 120min after drink ingestion | ☐☐☐/☐☐☐ [mmHg] |
| Baseline preoperative heart rate [1/min] | ☐☐☐ [1/min] |
| **After 1^st^ ingestion** |  |
| Heart rate [1/min] at 15min after drink ingestion | ☐☐☐ [1/min] |
| Heart rate [1/min] at 30min after drink ingestion | ☐☐☐ [1/min] |
| Heart rate [1/min] at 45min after drink ingestion | ☐☐☐ [1/min] |
| Heart rate [1/min] at 60min after drink ingestion | ☐☐☐ [1/min] |
| Heart rate [1/min] at 75min after drink ingestion | ☐☐☐ [1/min] |
| Heart rate [1/min] at 90min after drink ingestion | ☐☐☐ [1/min] |
| Heart rate [1/min] at 105min after drink ingestion | ☐☐☐ [1/min] |
| Heart rate [1/min] at 120min after drink ingestion | ☐☐☐ [1/min] |
| **After 2^nd^ ingestion** |  |
| Heart rate [1/min] at 15min after drink ingestion | ☐☐☐ [1/min] |
| Heart rate [1/min] at 30min after drink ingestion | ☐☐☐ [1/min] |
| Heart rate [1/min] at 45min after drink ingestion | ☐☐☐ [1/min] |
| Heart rate [1/min] at 60min after drink ingestion | ☐☐☐ [1/min] |
| Heart rate [1/min] at 75min after drink ingestion | ☐☐☐ [1/min] |
| Heart rate [1/min] at 90min after drink ingestion | ☐☐☐ [1/min] |
| Heart rate [1/min] at 105min after drink ingestion | ☐☐☐ [1/min] |
| Heart rate [1/min] at 120min after drink ingestion | ☐☐☐ [1/min] |
| **After 3^rd^ ingestion** |  |
| Heart rate [1/min] at 15min after drink ingestion | ☐☐☐ [1/min] |
| Heart rate [1/min] at 30min after drink ingestion | ☐☐☐ [1/min] |
| Heart rate [1/min] at 45min after drink ingestion | ☐☐☐ [1/min] |
| Heart rate [1/min] at 60min after drink ingestion | ☐☐☐ [1/min] |
| Heart rate [1/min] at 75min after drink ingestion | ☐☐☐ [1/min] |
| Heart rate [1/min] at 90min after drink ingestion | ☐☐☐ [1/min] |
| Heart rate [1/min] at 105min after drink ingestion | ☐☐☐ [1/min] |
| Heart rate [1/min] at 120min after drink ingestion | ☐☐☐ [1/min] |
| Urinary output/24 hours | ☐☐☐☐ [ml]  = [ml]/h |
| Total fluid volume intraoperative  (components) | ☐☐☐☐ [ml] |
| Fluid volume 24 hours postoperative  (components) | ☐☐☐☐ [ml] |
| Vasopressor intraoperative |  |
| Type | Ephedrine ☐ Norepinephrine ☐ |
| Amount | Ephedrine ☐☐ [mg]  Norepinephrine ☐☐☐ [ ] |
| Vasopressor 24 hours postoperative | yes ☐ no ☐ |
| Type | Ephedrine ☐ caffeine ☐ Red Bull® ☐  Date/Time:  Ephedrine ☐ caffeine ☐ Red Bull® ☐  Date/Time:  Ephedrine ☐ caffeine ☐ Red Bull® ☐  Date/Time:  Ephedrine ☐ caffeine ☐ Red Bull® ☐  Date/Time: |
| Volume | Ephedrine ………… [mg]  Caffeine cups  Red Bull® [ml] |
| Analgesic use intraoperative | ☐ Fentanyl  ☐ Remifentanil  ☐ Morphine [mg]  ☐ Perfalgan [g]  ☐ Novalgin [g]  ☐ |
| Analgesic use 24 hours postoperative | ☐ Morphine [mg]  ☐ Perfalgan [g]  ☐ Novalgin [g]  ☐ Ketamin [mg]  ☐ |
| Temesta® use 24 hours postoperative | ☐ [mg] |
| Additional drinks/peroral volume | Type:  Volume:  Date/Time:  Type:  Volume:  Date/Time:  Type:  Volume:  Date/Time:  Type:  Volume:  Date/Time |

| **Outcomes 2: recovery** | |
| --- | --- |
| First mobilization | Date:  Time: ☐☐ h ☐☐ min |
| Pain in recovery area (NRS) 1  Pain in recovery area (NRS) 2 | /10  /10 |
| Pain on ward (1. Schicht) (NRS) | /10 |
| Pain on ward (2. Schicht) (NRS) | /10 |
| Pain on ward (3. Schicht) (NRS) | /10 |
| Pain on ward (4. Schicht) (NRS) | /10 |
| Pain on ward (5. Schicht) (NRS) | /10 |
| Wound healing 4 weeks postop (see photo) | ☐ Normal  ☐ Delayed  ☐ Infection  ☐ Hypertrophic scarring |

| **Outcomes 3: flap complications** | |
| --- | --- |
| Did any flap complications occur? | yes ☐ no ☐ |
| Onset | Date:  Time: ☐☐ h ☐☐ min |
| Type of flap complication | Anastomosis: arterial problem ☐  Anastomosis: venous problem ☐  Flap necrosis ☐  Infection ☐  Hematoma ☐  Seroma ☐  Other (specify): ☐ _________________ |
| Was return to OR required? | yes ☐ no ☐ |
| Was flap preserved? | yes ☐ no ☐ |

| **Adverse Event** | |
| --- | --- |
| Did any adverse event occur during administration of Red Bull**®** Energy Drink? | yes ☐ no ☐ |
| If „Yes“, please provide the information in the adverse events (AE) and give details below:-Skip | Nervousness ☐  Anxiety ☐  Tremor ☐  Insomnia ☐  Nausea ☐  Tachycardia ☐  Arrhythmia ☐  Other (specify) ☐ |
| Onset after ingestion of Red Bull**®** Energy Drink (approximate value) | ☐☐☐ min |
| Volume of Red Bull**®** Energy Drink ingested (approximate value) | ☐☐☐ ml |

| Investigator’s signature: | Date of signature: |
| --- | --- |
